# Outcome of capacity building in mental health for well-being volunteers

**DOI:** 10.3389/fpsyt.2023.1205344

**Published:** 2023-07-17

**Authors:** Shivanand Yaresheemi, Aravind Raj Elangovan, Kavita Jangam, Padmavathy Doraiswamy, Manoj Kumar Sharma, Prabha S. Chandra

**Affiliations:** ^1^Department of Psychiatric Social Work NIMHANS, Bengaluru, Karnataka, India; ^2^Center for Addiction Medicine, National Institute of Mental Health and Neuro Sciences, Bengaluru, Karnataka, India; ^3^Department of Clinical Psychology, National Institute of Mental Health and Neurosciences, Bengaluru, Karnataka, India; ^4^Department of Psychiatry, National Institute of Mental Health and Neurosciences (NIMHANS), Bengaluru, Karnataka, India

**Keywords:** well being volunteers, capacity building, volunteer motivation, community mental health, volunteer services

## Abstract

**Introduction:**

Volunteering is any activity in which time is given to assist another individual, group, or organization. It assists people who want to get involved in philanthropic programs that help volunteers develop awareness and lead healthier personal and social lives. Several volunteers have received specialized training in the fields in which they volunteer, such as health, mental health, education, or emergency rescue. Volunteers are rendering intervention in different areas in mental health. They are providing psychosocial support to the individuals, groups, community, promoting mental health through conducting various mental health awareness programs in the community. There is a growing concern about mental health in India due to the inaccessibility of services. The National Institute of Mental Health and Neuro Sciences (NIMHANS) being a premier institute for mental health, is devising innovative approaches to mental health care to reach the unreachable. One such initiative was to build the capacity of volunteers in the community who are interested in working for the cause of mental health.

**Methods:**

The objective of this study was to evaluate the outcome of the well-being volunteer program. This study used a descriptive cross-sectional research design, wherein all the 136 trained well-being volunteers (WBVs) were included as the study sample. The data was collected from the volunteers who attended the WBV program, which was initiated by NIMHANS Centre for Well-being (NCWB) and the Department of Psychiatric Social Work NIMHANS. A questionnaire on the outcome of the Well Being Volunteers program was developed for the study, and the Volunteer Motivation Inventory scale was used to collect the data from the WBVs. SPSS software was used to analyze the data. Ethical clearance was sought from the Institute Ethics Committee of NIMHANS.

**Results:**

The WBV program enhanced volunteers’ knowledge of mental health and benefited the volunteers in their personal and social life. They were also able to implement a satisfactory level of mental health-related volunteer activities in the community.

**Conclusion:**

Results of present study and the available literature suggest that engaging in voluntary services improves mental health knowledge. WBV program has provided opportunity to Volunteers to participate in mental health delivery system at different levels.

## Introduction

Mental health problems are widespread in all countries. One in every eight people worldwide has a mental disorder. The prevalence of various mental disorders varies by gender and age. Anxiety and depressive disorders are the most common in both men and women. Mental, neurological, and substance use disorders were estimated to account for one in every ten DALYs (10.1%) worldwide in 2019. 5.1% of the global burden was attributed to mental disorders.

In all countries, the burden of mental disorders spans the entire life course, with early beginnings in childhood with developmental disorders and childhood behavioural issues and continuing into adulthood and old age with depressive and anxiety disorders (1). Significant gaps and imbalances in information and research, governance, resources, and services characterize mental health systems worldwide (2). At the same time, mental health services, skills, and funding seem to be in short supply. These gaps are significant because they can severely hinder a country’s response to mental health. Mental health and mental health systems worldwide state that mental health needs are high, responses are insufficient, and the availability of affordable essential psychiatric medications is limited, particularly in low-income countries (3).

Most people with diagnosed mental health problems go untreated. In all countries, gaps in service coverage are adversely affected by differences in care quality. Other health issues are frequently given preference over mental health, and community-based mental health care is constantly underfunded in mental health budgets. On average, countries invest less than 2% of their healthcare budgets in mental health. 71% of people with mental illness do not receive mental health services. Most countries spend less than 20% of their mental health budget on community mental health services. In middle-income countries, psychiatric hospitals still contribute to more than 70% of mental healthcare expenditures (3, 4). Many countries face severe shortages of mental health professionals. There are shortages of mental health nurses, psychiatric social workers, psychiatrists, psychologists, counsellors, and other paid mental health workers in India.

Around half of the world’s population lives in countries where only one psychiatrist serves a population of 200,000 or more (2, 4). In low-income countries, there is less than one mental health worker of any kind per 100,000 people. These countries face a health workforce crisis, and the scarcity of human resources and training is similarly overwhelming for mental health (5). Across all income groups, most mental health workers are nurses, who comprise 44% of the global workforce for mental health. In all income groups, there is a significant shortage of specialized mental health workers for children and adolescents (2, 3). The scarce skills are compounded because few non-specialist doctors, nurses, and clinical officers have been trained to recognize and treat patients with mental health conditions in primary health care. The health worker shortage poses many problems for achieving the health-related Millennium Development Goals. Few countries followed the job-shifting strategy, using qualified healthcare volunteers to seek treatment for health problems (6–8). In this background, there is a growing concern about mental health in India due to the inaccessibility of services. Community-based mental health care is consistently underfunded. With a lack of human resources in the mental health field, most community members cannot access health and mental health services. These volunteers can reach out to the unreached population in the community.

The available literature also emphasized the need for volunteering in the fields of health and mental health and their contribution to the well-being of the community. This study shows light on the available literature. Participation in formal volunteering has positive outcomes, higher psychological well-being, greater self-esteem, physical, cognitive activity, and lower depressive symptoms, fostering skills; improving the individual quality of life, helping make future careers helping a community (9–12). Volunteering for several reasons and multiple benefits, career enhancement, “giving back” to society, helping others, cope up with stressful situations (13–20). well-being, lower depression, lower mortality, a longer life span, lower level of dependency, personal satisfaction, gain work experiences and develop personally and socially, and cultivate their skills better mental health (11, 21–25). Volunteers are rendering interventions in different areas of mental health. They provide psychosocial support to individuals, groups, and the community, promoting mental health by conducting various mental health awareness programs in the community; skilled volunteers can potentially prevent mental health issues like psychological stress and distress. It is a beneficial method with a scarcity of qualified mental health professionals to resolve evolving mental health issues in the community.

The above literature highlights the importance of volunteers and volunteerism in the mental health fields. Therefore, we need a perspective that focuses on the volunteer and the changes one goes through while volunteering because it will help us design volunteer programs and promote them so they attract committed volunteers, who will then continue their service for a longer time for community development as well as to serve the needs of the needy people in the community. However, the use of volunteers in a tertiary hospital and its outcomes were not reported in the current literature. Thus, the current study focused on the outcome of one such Program run by a tertiary hospital through its Centre for Well-Being.

Due to the inaccessibility of services, there is a growing concern about mental health in India. NIMHANS, a premier institute in the mental health field, is devising innovative approaches to mental health care to reach the unreachable. One such initiative is creating volunteers in the community who are interested in working for the cause of mental health. The Department of Psychiatric Social Work, NIMHANS in collaboration with the Centre for Well-Being (NCWB), has taken this initiative to train the volunteers to provide curative, preventive, and promotive services in the community. The volunteers include teachers, IT professionals, youth, NGO workers, people in business, retired people, homemakers, etc.

The training program modules include different ways and approaches to identifying and treating mental illness, counselling skills; stress management; suicide prevention; school mental health, and positive mental health. A participatory methodology that includes brainstorming, group discussion, group activity, role play, videos, and field demonstrations is used to train volunteers. The workshops are conducted in different modules on Saturdays as half-day workshops. All the modules are covered in 4 months. After attending all the modules, the volunteers are certified as well-being volunteers and can provide mental health services under the guidance and supervision of professionals from the NIMHANS. The scope of the trained volunteers is not limited to the hospital-based curative component of psychosocial and rehabilitation care but also expands to preventive and promotive programs in schools, colleges, and the community.

In this background, the current study attempts to understand volunteers’ motivation to join the Program and evaluate their satisfaction with the Program and the output delivered by them.

## Methods

The Well-Being Volunteers (WBVs) program was initiated in 2016. A batch of 40–50 volunteers would be trained in mental health every year. The trained volunteers provide mental health interventions at curative, preventive, and promotive levels. In order to understand the outcome of the program in terms of the content and the implementation of the services. The current study would help consolidate the activities carried out for and by the WBVs.

The objective of this study was to evaluate the outcome of the Well-Being Volunteer program. This study used a descriptive cross-sectional research design, wherein all 136 volunteers who were trained in 4 batched of training program were included as the study’s sample. Out of these, 95 volunteers participated in the study.

**Table:** 

S. No	Reasons for iteration in sample size	Number of Samples
1	Actual sample	138
2	Volunteers who passed away	02
3	Number of volunteers who did not respond	41
		Male – 21
		Female – 20
4	Final sample size	95

Data were collected from the volunteers who attended the WBV program which was initiated by the NIMHANS Centre for Well-Being and the Department of Psychiatric Social Work at the National Institute of Mental Health and Neuro Sciences (NIMHANS).

A questionnaire to understand the outcome of the Well Being Volunteers program was developed for the purpose of the study. The content of the questionnaire included the knowledge level of the volunteers in the areas of mental health, stress management, suicide prevention program, basic counselling skills, identification of mental health disorders, satisfaction level of the content of the training program, and various activities carried out by the volunteers. The questionnaire was validated by the research guide and the faculty involved in the well-being volunteer program.

The Volunteer Motivation Inventory scale, (26) consisting of 10 domains that include Values, Recognition, Social Interaction, Reciprocity, Reactivity, Self-Esteem, Social, Career Development, Understanding, and Protective dimensions was used to understand the motivation of the volunteers.

### Reliability and validity of the scale

Cronbach’s Alpha Reliability Coefficients for the VMI Scales (27).

**Table:** 

Volunteer Motivation Inventory	
Scale	Alpha
Career development	0.7712
Recognition	0.6295
Social interaction	0.8082
Reciprocity	0.6962
Reactivity	0.7257
Self esteem	0.7128

An online form consisting of all the above tools was prepared and the same was mailed to all the trained WBVs. They were asked to respond within 3 weeks.

SPSS software was used to analyze the data. Descriptive statistics, including frequency distribution, central tendency measures, and deviation, were used to describe the variables. The McNemar-Bowker test was used to compare the scores of each item between pre and post-test. Ethical clearance was obtained from the Institute’s ethics committee (No. NIMH/DO/BEH.Sc.Div./2020–21, Dt: 06.07.20).

## Results

### Socio demographic profile of the participants

The mean age of the respondents was 43.5 years the SD is ±12.7with a range of 20 to 73 years. Overall, 65.3% of respondents were females, and 50.5% were graduates. 43.1% of respondents learned about the Well-Being Volunteer Program through their friends. 27.4% of respondents got to know about the program from social media, 21.1% of respondents through Well-Being Volunteers, and.8.4% from print media.

#### Table 1: education qualifications

Table 1 describes the education qualifications of the respondents: 50.5% were graduates and 49.5% had completed post-graduation.

**Table 1:** Education qualifications.

Sl. No	Variables	Category	*N*	%
1.	Education qualification	Graduation	48	50.05
		Post-graduation	47	49.5
		Total	95	100

#### Table 2: volunteer motivation

Table 2 describes the descriptive statistics of various subsections of the VMI inventory. The descriptive statistics results indicate that values were considered the most important motivation, followed by understanding and reciprocity, self-esteem, recognition, reactivity, social interaction, and motivations were perceived as being fairly important, with mean scores above the midpoint of 3.00. Social, protective, and career development exhibited the greatest amount of variation among volunteers.

**Table 2:** Descriptive of volunteer motivation inventory.

Sub section	*N*	Range	Minimum	Maximum	Mean	Std. deviation
Values	95	2.20	2.80	5.00	4.0	0.53
Recognition	95	3.60	1.20	4.80	3.22	0.62
Social interaction	95	4.00	1.00	5.00	3.20	0.83
Reciprocity	95	4.00	1.00	5.00	3.92	0.78
Reactivity	95	3.25	1.75	5.00	3.45	0.78
Self-Esteem	95	3.40	1.60	5.00	3.53	0.75
Social	95	3.80	1.00	4.80	2.63	0.85
Career development	95	3.50	1.00	4.50	2.71	0.66
Understanding	95	2.80	2.20	5.00	3.97	0.59
Protective	95	3.80	1.20	5.00	2.93	0.80

### Level of satisfaction on well being volunteer’s program

60 and 38.9% of the respondents reported that they were very satisfied and satisfied with the program content. 75.8% of respondents reported being highly satisfied with the program’s methodology.

### Knowledge on various dimensions of the training program

#### Table 3: “Knowledge on psychosocial competencies” among children before and after attending Program

**Table 3:** Knowledge on psychosocial competencies among children before and after attending program.

Knowledge on psychosocial competencies among children before attending WBVs program	Has your knowledge on psychosocial competencies among children improved after attending WBV training program								McNemar Bowker test	df	*p* value
	Not at all		To some extent		To great extent		Total				
	*N*	%	*N*	%	*N*	%	*N*	%			
Not at all	0	0	9	9.5	10	10.5	19	20	54.13	3	*p* < 0.001
Some extent	1	1.1	23	24.2	46	48.4	70	73.7			
To great extent	0	0	3	3.2	3	3.2	6	6.3			
Total	1	1.1	35	36.9	59	62	95	100			

Table 3 shows the results of the McNemar-Bowker test to find out the difference in the level of knowledge on psychosocial competencies before and after attending the Well-Being Volunteer training program. The results showed that the change in the level of knowledge was statistically significant (McNemar-Bowker test value =54.13, df = 3, *p* < 0.001). 48.4% of respondents reported that their knowledge level had increased from some extent to a great extent after the training program.

### Knowledge on stress management, basic counselling skills, and suicide prevention strategies before and after attending well-being volunteer program

The results showed that their improved level of knowledge in stress management was statistically significant (McNemar-Bowker test value = 60.74, df = 3, *p* < 0.001). 55.8% of respondents reported that their stress management knowledge level had increased from some extent to a great extent after the training program. The results showed that their improved knowledge of basic counseling skills is statistically significant (McNemar-Bowker Bowker test value =52.03, df = 3, *p* < 0.001). 46.3% of respondents reported that their basic counselling skills knowledge level had increased from some extent to a great extent after the training program.

64.2% of respondents reported they did not have knowledge of suicide prevention before attending the Program. 50.5% of the respondents reported that the Program significantly improved their knowledge of suicide prevention from some extent to a great extent after attending the WBV training program. 61.1% of the participants reported a significant improvement in their knowledge level in identifying mental health disorders and treatment.

### Programs on mental health and stress management carried out after the training program

46.4% of the respondents reported that they were able to conduct mental health-related Programs for children based on what they learned in the WBV training program. 53.6% of the respondents reported that they did not conduct mental health-related Programs for children.29.5% of the respondents reported that they conducted 1–5 programs. 7.4% of respondents reported that they conducted 6–10 programs. 6.3% of respondents reported that they conducted 16 and above programs.

40% of the respondents could conduct a stress management program based on learning in the WBV training program. 23.2% of the respondents conducted 1–5 programs. 8.3% of the respondents conducted 6–10 programs.

#### Table 4: organizing stress management program and volunteer motivation

Table 4 shows the results of the independent sample *t*-test to find out the difference in the mean score of the social dimension of the Volunteer Motivation Inventory between respondents who organized the stress management Program and those who did not.

**Table 4:** Organizing stress management program and volunteer motivation.

Organized stress management program	*N*	Social dimension		*t* value	df	*p* value
		Mean score	SD			
Yes	38	2.9	0.83	2.780	93	0.007 (*p* < 0.05)
No	57	2.4	0.81			

The results showed that there was a statistically significant difference (*t* = 2.780, df = 93, *p* < 0.05) in the mean score of the Social Dimensions of Volunteer Motivation Inventory between those who organized the Program (2.9 ± 0.83) and those who did not organize the Program (2.4 ± 0.81). There was no significant difference in the mean score of other dimensions of the Volunteer Motivation Inventory between those who organized the Program and those who did not organize the Program.

### Application of stress management strategies

97.9% of the respondents reported that the training program’s stress management module helped them manage stress at an individual level after attending the Program. 93.7% of the respondents reported that the stress management module helped them to manage stress at the family level after attending the Program. 76.8% of the respondents reported that the training program’s stress management module helped Well-Being Volunteers manage stress levels at the workplace.

### Referrals

90.5% of the respondents reported that the WBV training program helped identify and refer persons with mental health.

Problems to NIMHANS, NCWB, and other mental health professionals.

71.6% of the respondents reported that they made referrals for 1–10 persons. 6.3% of respondents reported referrals for 11–20 persons. 2.1% reported 21–30 referrals. 20% of respondents reported no referrals.

### Involvement in patient care activities of NIMHANS

27.4% of the respondents reported being involved in patient care activities in the inpatient care services of NIMHANS. 22.1% of the respondents reported being involved in outpatient care activities of NIMHANS.

## Discussion

**Socio-demographic profile of the participants**: in the present study, the majority of the respondents are females compared to males. Results of one more study also corroborate this finding, which indicates that females are more active in volunteering activities than males, which is corroborated with other similar findings that females are more active in volunteering compared to men (28). The educational level in the results indicates that this study’s results corroborate previous studies that found that literacy was significant in predicting knowledge and participation in volunteering (29). The above study supports the present study findings that show that the majority of the volunteers who attended the program finished as graduates, which shows that having literacy makes significant contributions to volunteering.

**Training methodology**: the research study suggested that rather than more formal lecture-style approaches, teaching should concentrate on doing through role playing. This strategy was aimed at non-professionals with little formal experience (30). The above study supports the findings of the present study, where all the respondents showed satisfaction with the participatory methodology used in the Program such as role play, group activities, group discussions, games, and case discussions. The participatory methodology allowed them not only to actively participate during the session but also to get involved in every bit of it and internalize the skills discussed in each module.

**Benefits of volunteering**: volunteering contributes to a person’s physical, social, and emotional well-being. The International Association of Volunteer Efforts (IAVE) is continuously making efforts to join volunteers and motivating volunteers to conduct activities in the community, giving importance to rendering services for needy people. It is working in the areas of mental health knowledge development, leadership, and advocacy. Volunteering can have mental health effects, especially for the elderly (9, 20). Volunteering helps people have access to physical and psychological assets (19, 23, 24). Voluntary work leads to greater health benefits and helps people cope with stressful emotions, including depression and anxiety; it also leads to greater life satisfaction, and more successful accomplishment of tasks (25, 28–33). The above studies support the present study, where volunteers actively participated in the WBV training program to improve their knowledge of mental health and were interested in rendering services in the mental health field in the community. This Program enhanced the knowledge of volunteers on mental health and benefited them in their personal and social lives. It has also helped in the early identification of mental health problems and addressing the issue through various modes in the community, such as referral services, counselling services, stress management program at work, and suicide prevention awareness program.

**Enhancement of mental health knowledge**: in the current study, it is found that there is an increase in volunteers’ knowledge and level of understanding in areas related to chronic psychiatric illness, but the efficacy of anti-stigma approaches after 4 weeks of improvement in terms of increasing awareness of mental health and decreasing stigmatising behaviours was low, highlighting the need for more comprehensive interventions that look at long-term effects as well as the usage of booster interventions for long-term survival (34, 35). Similar to the above studies on the involvement of volunteers in mental health awareness program to reduce stigma and discrimination in the community, the majority of well-being volunteers conducted various mental health awareness programs in community, such as mental health stalls, stress management, suicide prevention programs, awareness activities like street play, organized anti-stigmatisation awareness programs etc. These activities created awareness in the community, motivated people to get proper treatment for mental illness, and secured the rights of those with mental illness.

### Motivation and volunteering

A comparison between volunteer motivation and the activities carried out by the volunteers in the current study shows that the Social Dimensions of Volunteer Motivation Inventory had some influence on the volunteering activities carried out by the volunteers. The variables in the social dimension clearly mention the volunteer’s contacts through which they joined the Program. So, the difference could be due to the fact that those who scored higher on social dimensions participated more in volunteering activities compared to those who scored lower. The volunteering activities are initiated by the volunteers immediately after completing the training program; they chose their area of interest and performed the volunteering activities in that respective area effectively. For example, volunteers who were working with children in schools and colleges were able to do more child-related programs.

Volunteers who were in the IT sector were able to organize and conduct programs related to stress management. Volunteers who were interested in patient care activities were more involved in providing care for people with mental illness. So, the Well Being Volunteers program has given a wider scope for volunteers to choose their area of work, followed by the training program and the continuous supervision by the trainers has also helped them plan the activities effectively.

### Integration of mental health component in volunteer training

From current study it is very evident that involvement of volunteers in mental health training and providing services is helped in addressing the gap between the demand and available mental health resources. So, inclusion of similar mental health contents in the existing volunteer training programs organized by various Government and Non-Governmental organizations at national and international level would help in successful integration of mental health component in the training. This will ensure effective delivery of promotion of mental health services and prevention of mental disorders at community level.

## Conclusion

Volunteers have a major role in the field of mental health care. Well-being Volunteer program bridge the gap between mental health professionals and those who are in need of mental health services. Their volunteer services will make a significant impact in early identification and referral of persons who are in need of mental health services. Volunteers are also instrumental in conducting mental health awareness programs, stress management programs and suicide prevention programs at schools, colleges and workplaces. This will help in dealing with stigma and discrimination against mental illness in an effective manner. The current study has helped to understand the outcome of such volunteer initiatives in the field of mental health. Such programs would be of great support in strengthening the mental health delivery systems in countries where there is a resource crunch.

### Limitations of the study

- The outcome of the volunteer program discussed in the current paper is restricted to urban settings.
- The qualitative details of the program such as the content of the programs carried out by volunteers and feedback of the participants are not included in the current study.

## Data availability statement

The original contributions presented in the study are included in the article/Supplementary material, further inquiries can be directed to the corresponding author.

## Ethics statement

The Department of Psychiatric Social Work, NIMHANS Institute sub-ethics committee provided ethical approval. The patients/participants provided their written informed consent to participate in this study.

## Author contributions

SY, AE, KJ, PD, MS, and PC contributed to conception and design of the study. SY wrote the first draft of the manuscript. AE and KJ performed the statistical analysis. PD, MS, and PC wrote sections of the manuscript. All authors contributed to the article and approved the submitted version.

## Conflict of interest

The authors declare that the research was conducted in the absence of any commercial or financial relationships that could be construed as a potential conflict of interest.

## Publisher’s note

All claims expressed in this article are solely those of the authors and do not necessarily represent those of their affiliated organizations, or those of the publisher, the editors and the reviewers. Any product that may be evaluated in this article, or claim that may be made by its manufacturer, is not guaranteed or endorsed by the publisher.
