# Supplementary material for: Outcome of capacity building in mental health for well-being volunteers
Source: Front Psychiatry. 2023 Jul 17;14:1205344. doi: 10.3389/fpsyt.2023.1205344 (PMC10390059; doi:10.3389/fpsyt.2023.1205344)
Supplement: Supplementary file 1 [file Table_1.DOCX]

| **Table 01: Descriptive of Volunteer Motivation Inventory:** |
| --- |

| **Sub section** | **N** | **Range** | **Minimum** | **Maximum** | **Mean** | **Std. Deviation** |
| --- | --- | --- | --- | --- | --- | --- |
| **Values** | 95 | 2.20 | 2.80 | 5.00 | 4.0 | .53 |
| ***Recognition*** | 95 | 3.60 | 1.20 | 4.80 | 3.22 | .62 |
| ***Social Interaction*** | 95 | 4.00 | 1.00 | 5.00 | 3.20 | .83 |
| ***Reciprocity*** | 95 | 4.00 | 1.00 | 5.00 | 3.92 | .78 |
| ***Reactivity*** | 95 | 3.25 | 1.75 | 5.00 | 3.45 | .78 |
| ***Self-Esteem*** | 95 | 3.40 | 1.60 | 5.00 | 3.53 | .75 |
| ***Social*** | 95 | 3.80 | 1.00 | 4.80 | 2.63 | .85 |
| ***Career Development*** | 95 | 3.50 | 1.00 | 4.50 | 2.71 | .66 |
| ***Understanding*** | 95 | 2.80 | 2.20 | 5.00 | 3.97 | .59 |
| ***Protective*** | 95 | 3.80 | 1.20 | 5.00 | 2.93 | .80 |

**Table 2: Knowledge on psychosocial competencies among children before and after attending Program**

| **Knowledge on psychosocial competencies among children before attending WBVs Program** | **Has your knowledge on psychosocial competencies among children improved after attending WBV training Program** | | | | | | | | **McNemar**  **Bowker**  **test** | **df** | **p value** |
| --- | --- | --- | --- | --- | --- | --- | --- | --- | --- | --- | --- |
|  | **Not at all** | | **To some extent** | | **To great extent** | | **Total** | |  |  |  |
|  | N | % | N | % | N | % | N | % | 54.13 | 3 | P<0.001 |
| **Not at all** | 0 | 0 | 9 | 9.5 | 10 | 10.5 | 19 | 20 |  |  |  |
| **Some extent** | 1 | 1.1 | 23 | 24.2 | 46 | 48.4 | 70 | 73.7 |  |  |  |
| **To great extent** | 0 | 0 | 3 | 3.2 | 3 | 3.2 | 6 | 6.3 |  |  |  |
| **Total** | 1 | 1.1 | 35 | 36.9 | 59 | 62 | 95 | 100 |  |  |  |

**Table 3: Organising Stress management Program and Volunteer Motivation**

| **Organized stress management program** | **N** | **Social Dimension** | | **t Value** | **df** | **p value** |
| --- | --- | --- | --- | --- | --- | --- |
|  |  | **Mean Score** | **SD** |  |  |  |
| Yes | 38 | 2.9 | 0.83 | 2.780 | 93 | 0.007  (p<0.05) |
| No | 57 | 2.4 | 0.81 |  |  |  |
